# Design and control of soft biomimetic pangasius fish robot using fin ray effect and reinforcement learning

**DOI:** 10.1038/s41598-022-26179-x

**Published:** 2022-12-18

**Authors:** Samuel M. Youssef, MennaAllah Soliman, Mahmood A. Saleh, Ahmed H. Elsayed, Ahmed G. Radwan

**Affiliations:** 1 Bio-Hybrid Soft Robotics Laboratory (BHSRL), Nile University, Sheikh Zayed City, 12588 Egypt; 2 Innovation Hub, Nile University, Sheikh Zayed City, 12588 Egypt; 3 Department of Engineering Mathematics and Physics, Cairo University, Giza, 12613 Egypt; 4 Nanoelectronics Integrated Systems Center (NISC), Nile University, Sheikh Zayed City, 12588 Egypt

**Keywords:** Mechanical engineering, Computer science

## Abstract

Soft robots provide a pathway to accurately mimic biological creatures and be integrated into their environment with minimal invasion or disruption to their ecosystem. These robots made from soft deforming materials possess structural properties and behaviors similar to the bodies and organs of living creatures. However, they are difficult to develop in terms of integrated actuation and sensing, accurate modeling, and precise control. This article presents a soft-rigid hybrid robotic fish inspired by the Pangasius fish. The robot employs a flexible fin ray tail structure driven by a servo motor, to act as the soft body of the robot and provide the undulatory motion to the caudal fin of the fish. To address the modeling and control challenges, reinforcement learning (RL) is proposed as a model-free control strategy for the robot fish to swim and reach a specified target goal. By training and investigating the RL through experiments on real hardware, we illustrate the capability of the fish to learn and achieve the required task.

## Introduction

Underwater depths have proven to be very challenging environments for humans to venture into. Researchers and engineers strive to build underwater robotic systems to accomplish this dangerous endeavor. From oceanic investigation and marine life exploration to execution of underwater missions and sample gathering to monitoring and maintenance of offshore and underwater structures, many complex tasks need to be done in harsh unpredictable conditions. These aquatic tasks are commonly carried out using underwater vehicles such as remotely operated vehicles (ROVs) and autonomous underwater vehicles (AUVs). However, AUVs are currently limited to mid-depth exploratory operations, while ROVs are more suitable for deep seabed investigation but are constrained by the requirement of tethering and manual piloting. In addition, these systems are mainly made from rigid parts that limit their maneuverability, their ability to safely interact with their surroundings, and their adaptability to the unforeseeable aquatic climate^1^. Leveraging the new technological advancements in biomimetics and soft robotics provides promising solutions to build robotic systems capable of operating more naturally and withstanding these harsh environments^2,3^.

Studying the various biological marine creatures offers insights into the characteristics allowing them to live in and populate vast oceanic regions. Taking inspiration from the morphologies of underwater living organisms, their techniques for swimming and locomotion, and their sensory capabilities aids in the development of bioinspired robotic systems similar to these creatures, making these robots more suitable for underwater applications. Swimming motion amongst underwater creatures shows a variety of locomotion techniques, guided by the morphological structures and shapes of such creatures^4^. The majority of aquatic creatures possess compliant bodies and rely on their body deformation to generate the thrust needed for locomotion. The most common classification of fish swimming motion has been adopted according to fish anatomy and their propulsors^5^. By moving their body and fins with different undulating or oscillatory frequencies, fish can generate the thrust needed for forward motion, turning, and escape maneuvers. While this classification is mainly concerned with fish and batoids swimming, other marine creatures such as jellyfish, turtles, echinoderms, and crustaceans use different types of locomotion like jet propulsion, drag-induced swimming, and crawling. In addition, several studies focused on fish’s individual and group behaviors, and their social interactions with biomimetic fish robots^6–10^. These investigations provide insight into the use of fish-like robots to interact with and study fish behavior, and mechanisms responsible for mixed phenotype aggregations, as well as, provide biohybrid stimuli for further social analysis such as anxiety treatment and information transfer.

The field of soft robotics offers successful approaches for building bioinspired robotic systems in general^11–14^, and more specifically robots inspired by biological marine creatures^15^. The use of soft materials to develop robots with compliant bodies and large degrees of freedom can take us a step closer to mimicking marine creatures with complex locomotion^16^. Several attempts have been made to exploit their deformability to design biomimetic soft robots capable of imitating biological swimming motion^17^. One approach made use of hydraulic elastomers to develop a soft robotic fish capable of performing several swimming maneuvers^2^. Alternatively, a bioinspired robotic fish uses ionic polymer-metal composite (IPMC) actuators as the pectoral and caudal fins^18^. Another team was able to mimic the cephalopod molluscs by using hydraulic smart soft bending actuators to build the tentacles that aid the cephalopod in maneuvering^19^. A brittle star-inspired soft robot uses twenty shape memory alloy (SMA) wires to actuate five flexible legs and perform underwater crawling^20^. A robotic jellyfish uses the fin ray structure to mimic the soft tentacles of the jellyfish and their motion^21^. Some of these soft robots were tested in real underwater environments, such as the snailfish robot actuated using dielectric elastomers (DEs), which was able to operate at great depths^3^.

One of the biggest challenges in soft robotics is the modeling and control of these non-linear complex systems^22^. Research has been tackling these challenges using various approaches^23^. Some approaches rely on model-based control techniques, however, developing these techniques has proven to be a difficult task due to the complexity of developing models for high-dimensional soft robotic systems^24^. Several modeling theories, approximation models, and dimensionality reduction techniques are proposed to simplify the modeling task. By contrast, model-free control does not require a model or prior information about the system but relies mainly on the input-output behavior collected directly from the system to learn an approximate representation of it. Reinforcement learning (RL) is one of these model-free control techniques that have been providing promising results in recent years^25^. RL is a data-driven learning process that depends on having the agent interact with its environment by taking certain actions and observing its new state. The agent is then given a reward based on the task it needs to complete and the RL algorithm learns a policy to map the state-action pairs.

In particular, RL has been implemented for soft robotics control in general and specifically in the case of underwater soft robotics^26^. One group used a Q-learning algorithm with experience replay to maximize the swimming speed of a cuttlefish soft robot actuated by a DE membrane^27^. Soft actor-critic (SAC) was also used to learn a control strategy for a robotic eel with compliant bodies to allow it to swim efficiently in a straight line^28^. SMAs were also used to actuate a soft robot by employing Q-learning to develop a control policy for end effector locomotion^29^. Additionally, an octopus-inspired soft robot used deep q learning (DQN) to control the posture of the soft arms of the robot^30^. One approach used a deep deterministic policy gradient (DDPG) algorithm to learn a control policy for soft continuum arms^31^. However, training RL agents is a costly process in terms of computation time and resources, and it becomes more complex for soft robots due to their non-linear dynamics and elastic properties. To solve this problem, a research group implemented an RL method that ignores the soft materials’ properties and structure of the robot, and it was applied to the Honeycomb PneuNets soft robot^32^. A different technique to simplify the RL process is the use of learning from demonstration (LfD) and imitation learning methods, such as in the case of the STIFF-FLOP robotic arm^33^, where the movement patterns of an octopus arm were transferred to the robot arm as a guide to speed up the learning process. Combining a model with RL algorithms could help the policy learning process. One research proposes a model-based RL for closed-loop control of soft robotic manipulators^34^. The proposed approach uses a recurrent neural network (RNN) to learn the forward dynamic model, which is then used to develop a closed-loop predictive controller. The mentioned studies investigated the use of different RL algorithms for soft robotics control, including combining it with imitation learning. However, the success of RL in high-level tasks of soft robots like underwater navigation is still a question. In addition, comparisons between several RL algorithms’ performances for the same task have not been discussed.

**Figure 1:**
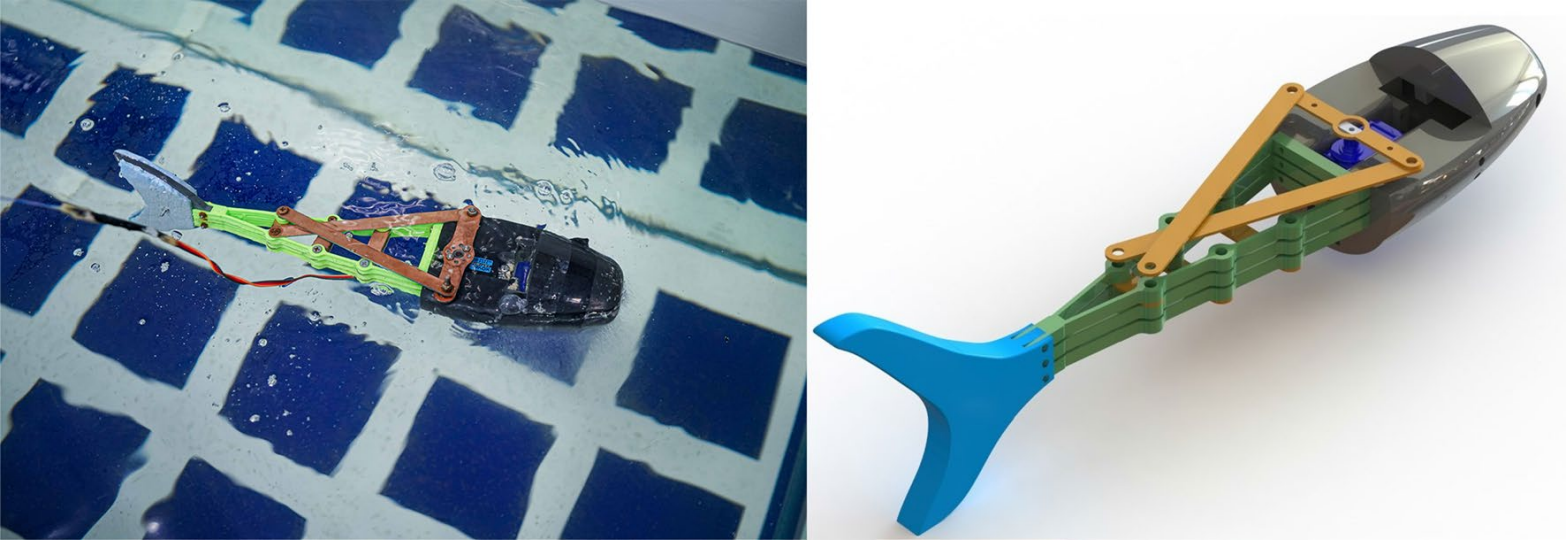
Soft biomimetic Pangasius fish robot. Robot prototype (left). Complete CAD of the robot (right).

In this paper, we propose a design for a biomimetic fish robot inspired by the Pangasius fish, using the fin ray effect (FRE) for soft body actuation to mimic the fish’s body and tail undulation (Fig. 1). We investigate the use of three RL algorithms to teach the robot to swim to a specific goal. By achieving the task of underwater navigation and reaching target goals, the biomimetic robot fish developed in this work could be integrated into actual aquatic environments in the future. The main application of such robots is performing underwater exploration, researching marine life, monitoring coral reefs, and gathering samples without disturbing or destroying the environment. Such research is important to study the change in the underwater ecological system and the effect of climate change on it, giving insight into the needed actions to mitigate this effect.

## Materials and methods

### Pangasius fish morphological analysis

To build a soft underwater robot that mimics fish locomotion, an actual fish is studied through visual motion analysis to obtain insight and parameters relevant to the design and control of the equivalent biomimetic fish robot. The Pangasius fish was recorded using a webcam over several periods to obtain videos of its swimming motion. There are two main approaches to performing motion capture and tracking, marker-based and markerless tracking^35^. The traditional marker-based methods rely on having markers at the different points that will be tracked. These markers could be retro-reflective parts attached to the subject being tracked or differently colored parts, or in some cases, inertial measurement units are embedded and their data help estimate the motion of certain points. Such methods require hardware preparations but no further annotations are needed. On the other hand, The markerless tracking methods depend on having labeled ground truth done by humans, then machine learning models such as convolutional neural networks (CNNs) and residual networks (ResNets) are trained to estimate the motion based on the labeled keypoints.

**Figure 2:**
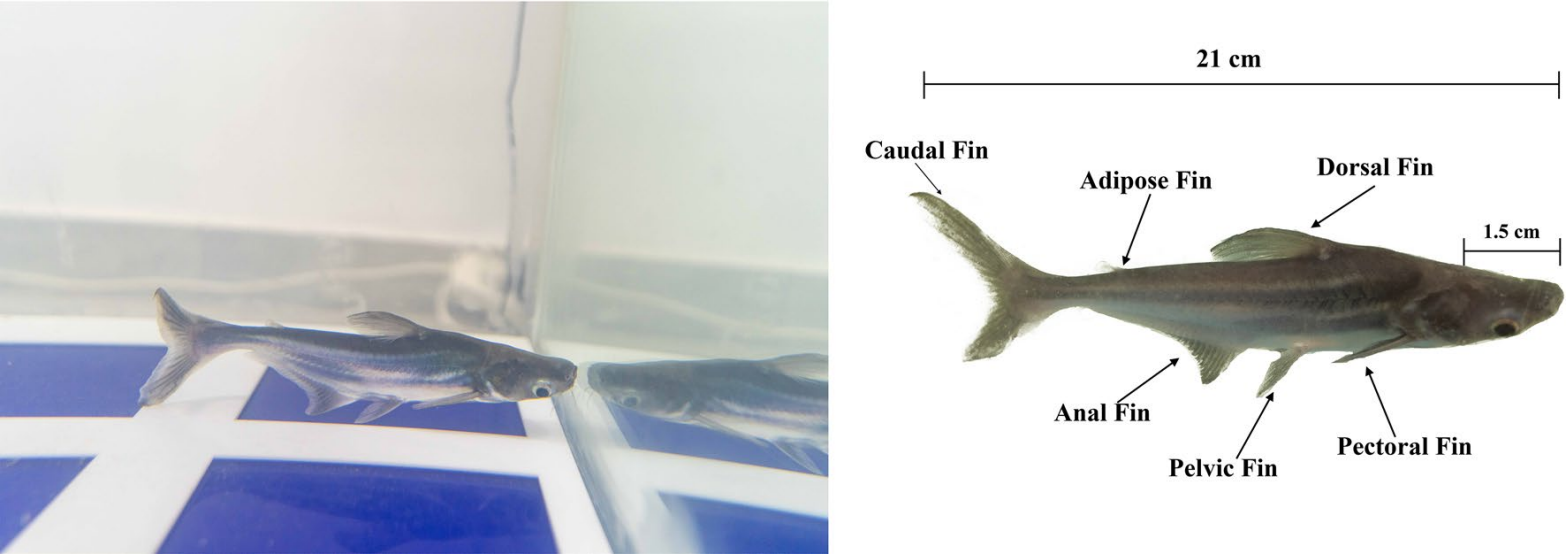
Real Pangasius fish anatomy captured by image processing motion system that presents the real dimension and morphological structure for the fish.

To perform motion analysis for this experiment, the markerless approach was used by preparing a motion capture setup. It is comprised of a water tank of dimensions 120 $\times$ 70 cm. The fish recorded for the analysis is from the Pangasius genus, a freshwater class of medium to very large shark catfishes. The dimensions of the fish in the study are roughly 21 cm in total body length, 2.5 cm in width, and 1.5 cm of head length “Lf”^36^. The fish has seven fins across its body, as shown in Fig. 2, two pectoral fins, a dorsal fin, a pelvis fin, an anal fin, an adipose fin, and a caudal fin at its tail end^37^. The data acquisition is carried through a Logitech C920 visual monocular camera. The camera was mounted on top of the tank to provide a full top view of the tank’s area. Video streams of the fish’s swimming sequence were captured at 30 frames per second (fps) using the camera.

#### Pose estimation

Studying the swimming patterns and motion of the fish requires performing pose estimation on the recorded video data to track the deformation and motion of the fish’s different body parts during its swimming sequences. The pose estimation step was performed using DeepLabCut, a deep learning platform for markerless animal pose estimation^38^.

**Figure 3:**
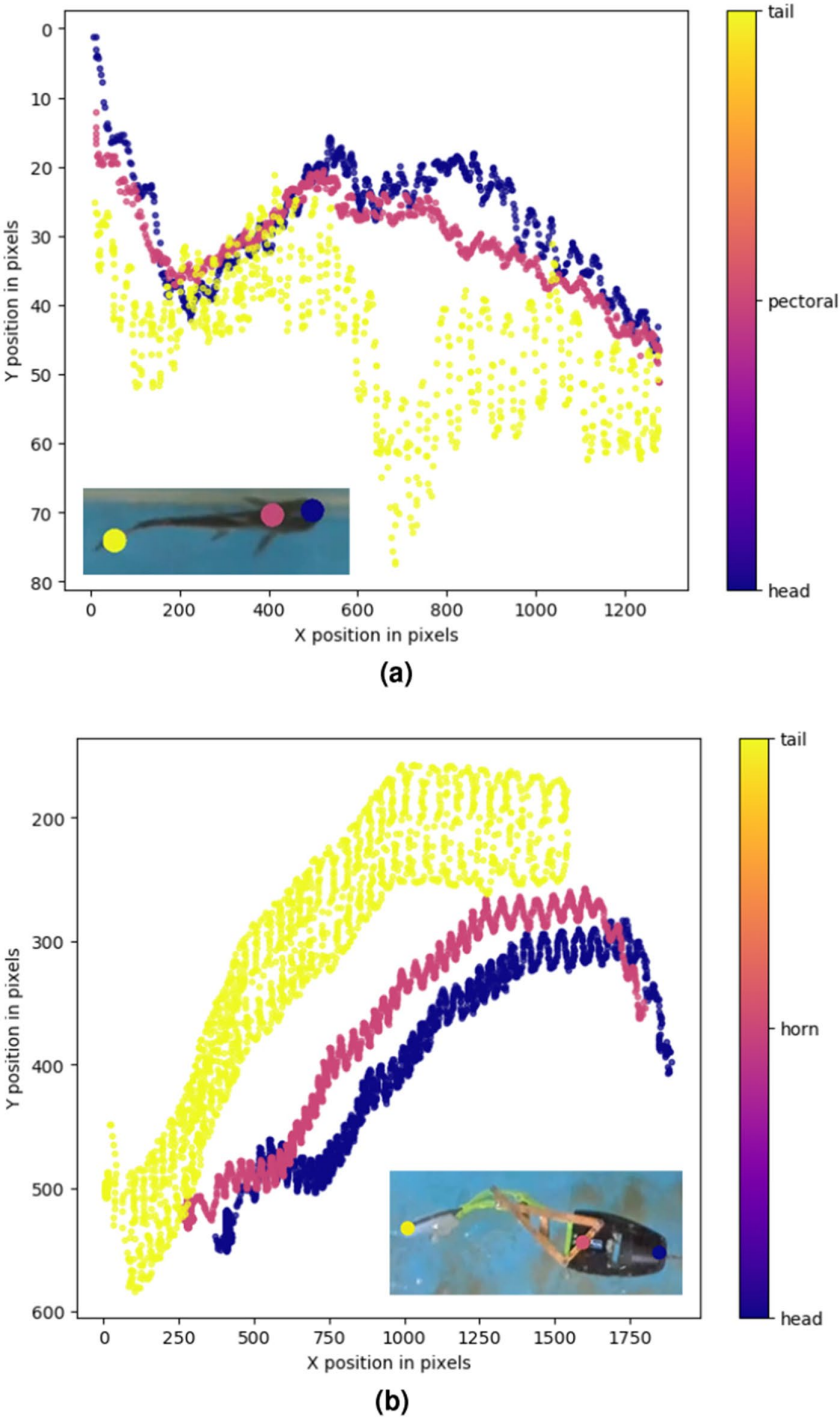
Results of the pose estimation and trajectory plot for both the fish and the robot. (**a**) Fish pose estimation. (**b**) Robot pose estimation.

During the pose estimation process (Fig. 3), three body parts of the fish are defined to be tracked: the head, the center of the pectoral fins, and the caudal fin. Several samples were taken from the captured videos and annotated with the body parts. A ResNet neural network with 152 layers is trained using the video streams to estimate the position of these points. The network was trained for 200,000 epochs reaching training and testing errors of approximately 3 and 6 pixels, respectively.

#### Swimming analysis

After obtaining the positions of the needed body parts, further analysis is conducted on the predicted pose of the fish to investigate the important parameters responsible for the swimming motion of the fish, which should help design the biomimetic fish. Such crucial parameters include the fish’s tail frequency and amplitude, and the resulting velocity at which the fish can swim due to its undulating motion. During carangiform swimming, the locomotion relies mostly on the undulation motion of the body and caudal fin, while the pectoral, pelvic, and dorsal fins help the fish balance and swim up and down.

By analyzing several sample videos of the fish’s swimming, the tracking of the tail’s motion allows us to obtain the undulation frequency of the fish’s body. While stationary, the frequency of the tail ranged from 0.7 to 2 hertz (Hz). During low-speed swimming, the frequency ranged from 1 to 2.5 Hz, while it reached up to 4.5 Hz during high-speed swimming. The attained speed during low-speed swimming was in the range of 5–6 cm/s, and up to 65 cm/s for high-speed swimming.

### Soft-rigid biomimetic pangasius fish robot design and prototyping

**Figure 4:**
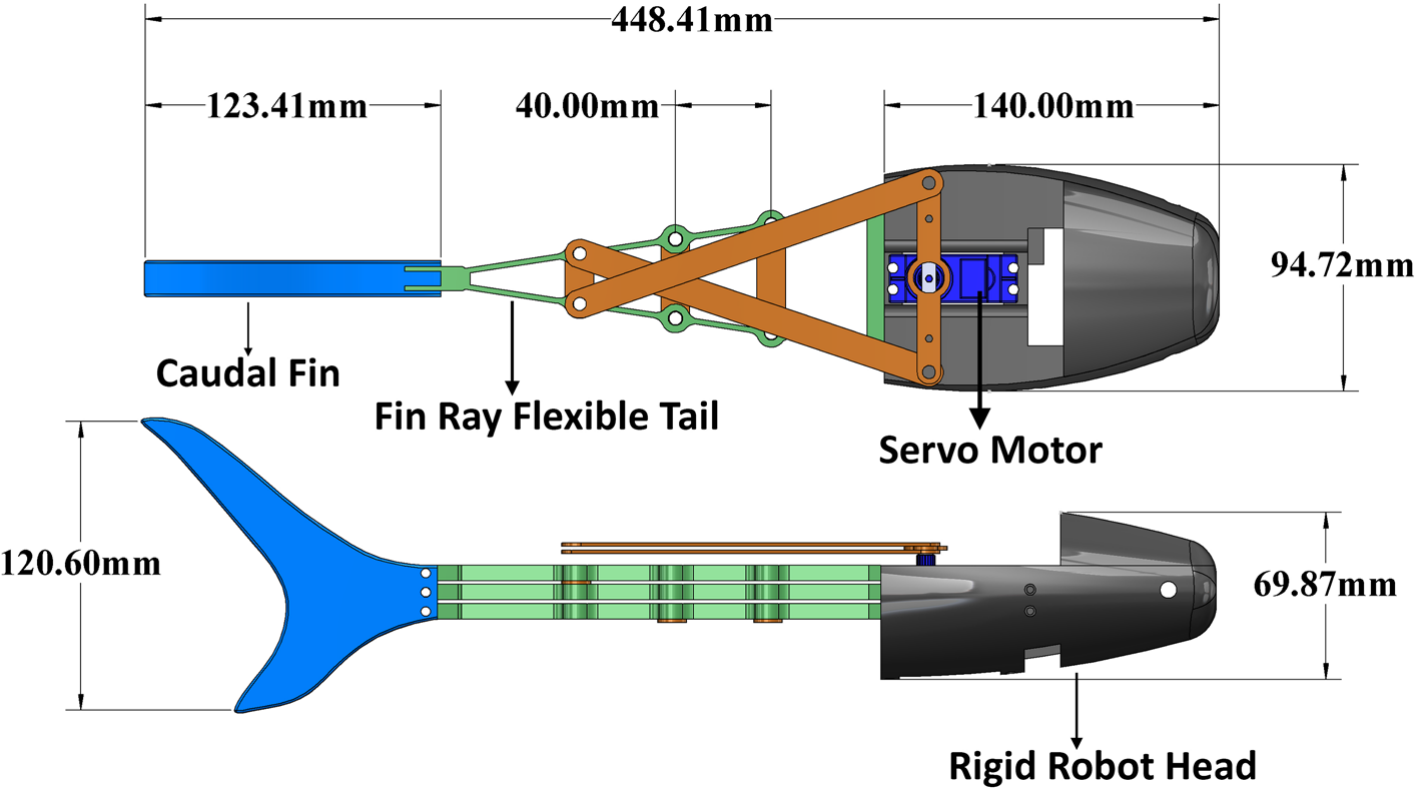
Detailed design and dimensions of robot design with an approximate double scale of real fish dimensions.

The soft-rigid biomimetic Pangasius fish is designed based on the dimensions of the real fish that is captured using the vision system, as described previously. The robot’s dimensions are scaled to double the dimensions of the real fish. The design is distributed into three main parts: fish rigid head, fish flexible tail, and caudal fin, as shown in Fig. 4. The fish’s body is responsible for the undulatory motion needed to move in the water. A soft tail is designed to mimic the fish’s tail and its motion, based on the fin ray effect (FRE), which is inspired by the tail fins of fish, making it a suitable option to provide similar swimming motion^39^. The fin ray compliant structure is chosen due to its high similarity to the naturally efficient bony tail of the fish. The actuator relies on a simple compressive force that causes the fin ray to bend in the direction of the force, deforming the tail into a concave shape that encloses a large volume of water and then accelerates it toward the rear. This motion results in a highly efficient forward thrust of the robot, analogous to fish swimming.

The fin ray consists of a flexible outer body, rigid links between its segmentation, and rigid connections between the servo motor (the source of actuation) and the actuating points in the fin ray itself. The actuation is driven by a waterproof servo motor that applies force to the sides of the fin ray making it bend in the direction of the force. By rotating in an oscillatory motion, the servo applies force on both sides of the actuator, working as an underactuated mechanism and resulting in the needed undulatory motion. However, the limited torque of the actuation servo motor makes it hard to generate enough thrust to perform fast maneuvers and the possible tail beat frequencies are also limited by the servo’s speed. In addition, the absence of pectoral fins on the robot limits the ability to swim with stable forward motion.

Different manufacturing techniques based on additive manufacturing are followed to produce the biomimetic Pangasius robot^40^. Due to the high complexity of the fish head part, selective laser sintering (SLS) is selected for production using the Sinterit Lisa Pro. The material used for SLS printing is PA12 Smooth, a Nylon-based material that is selected for its high durability. For the flexible fin ray, a high hyperelastic material is needed for its construction due to the low complexity of the design. The fused deposition modeling (FDM) 3D printer is selected to manufacture this part using the Felix 4Tec with a flexible material: the Extrudr FLEX medium material. Finally, a high-rigidity material is needed for rigid links and rigid connectors to withstand the tension forces exerted by the servo motor. The material being used is a glass-reinforced epoxy laminate material (FR-4) and is cut through a CO2 laser machine.

**Figure 5:**
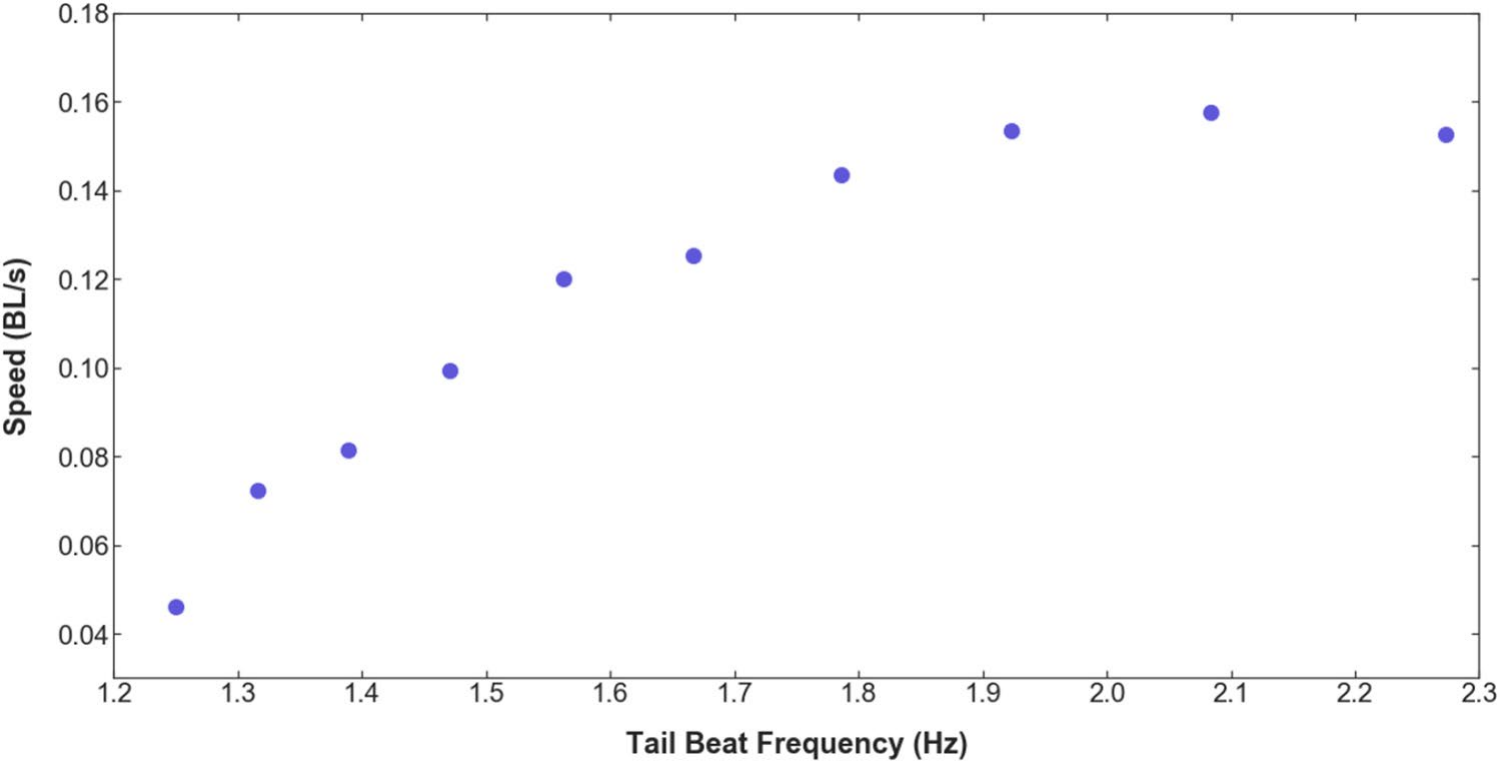
Robot’s swimming speed versus tail beat frequency.

**Figure 6:**
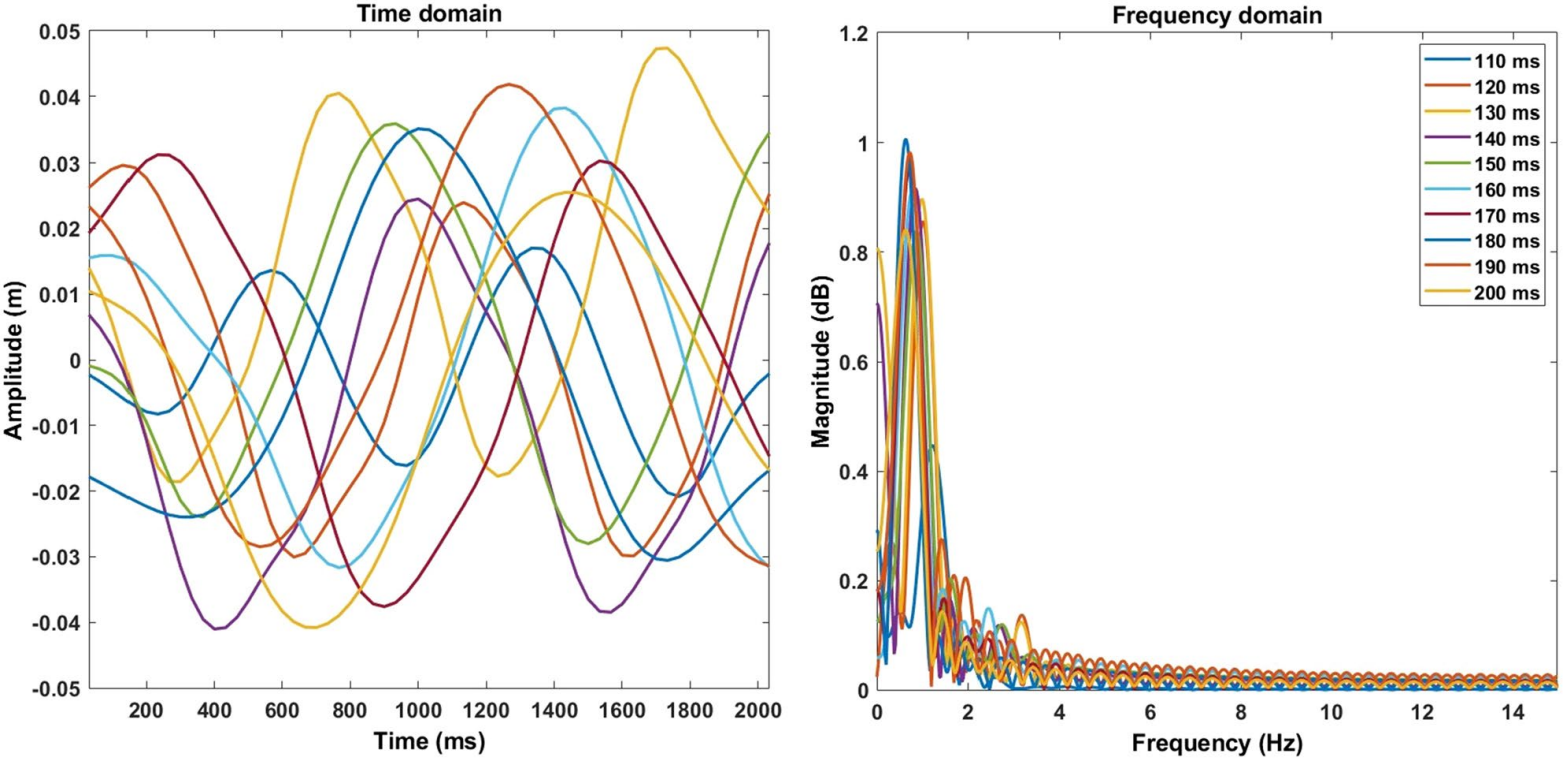
Time domain and frequency domain analysis of the swimming speeds of the robot. Each signal represents a swimming speed defined by the time to perform a single tail stroke in ms, ranging from 110 to 200 ms. The time domain shows the amplitudes of the tail’s undulatory motion, while the frequency domain shows the dominant frequency at each speed.

The robot’s swimming is achieved mainly through the undulation of the soft fin ray tail. By keeping the frequency of the undulation constant, the robot is able to swim forward, and depending on whether the frequency is low or high would make the robot swim slower or faster, respectively. However, performing a sequence of different frequencies in succession would cause the fish to change direction. In addition, the effect of the fluid perturbation and its interaction with the robot also affects its swimming. The robot’s swimming performance is assessed using the same method as the fish. By tracking the robot’s head and tail, the swimming speed and the tail beat frequency are obtained. We observe that the relation between the robot’s swimming speed, represented as the fish’s body length per second (BL/s), and its tail beat frequency (Hz) is similar to a second-order polynomial, as shown in Fig. 5. By applying Fourier transform to the robot’s tail motion at various speeds, the dominant undulatory swimming frequencies of the robot can be seen in Fig. 6.

**Figure 7:**
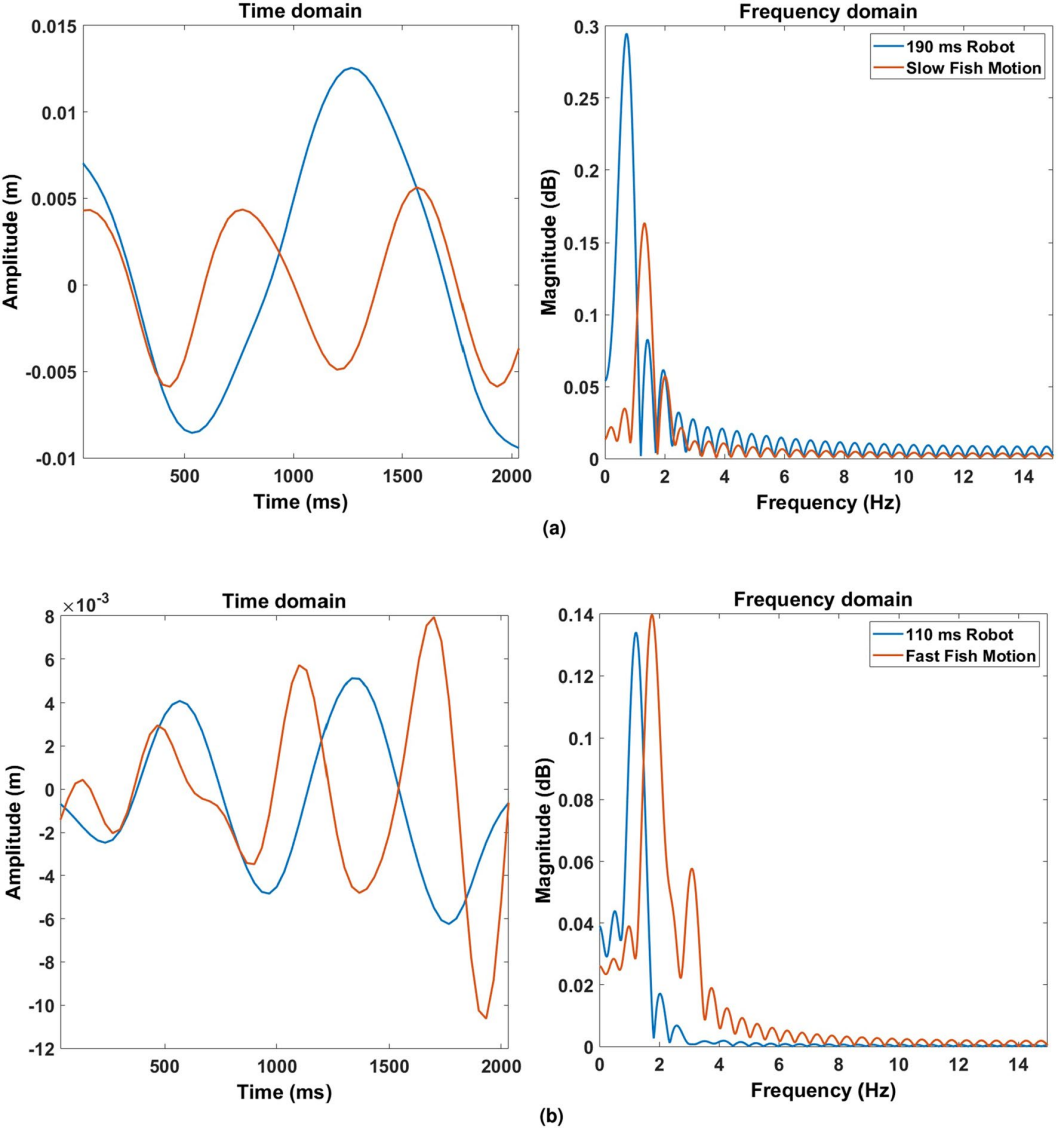
Comparison between the fish and robot tail beat frequencies. (**a**) Frequencies during slow swimming (robot’s tail stroke time at 190 ms). (**b**) Frequencies during fast swimming (robot’s tail stroke time at 110 ms).

Comparing the fish’s swimming to the robot’s shows the similarity between their tail beat frequencies at slow and fast swimming, shown in Fig. 7. The robot’s tail oscillation amplitude is scaled by the ratio between the robot and fish dimensions, to take into account the difference in their bodies’ deflections. The robot’s tail beat frequencies were chosen to be within the same range of the fish’s slow swimming, from 1 to 2.5 Hz. In addition, the cost of transport (COT) of the robot was calculated as an indication of its locomotion efficiency. COT is defined as the energy required to move a unit mass a unit distance^41^, according to the following equation:

1 $$\begin{aligned} COT = \frac{E}{mgd}= \frac{P}{mgv} \end{aligned}$$

where *E* is the robot’s input energy, *m* is the robot’s mass (0.422 kg), *g* is the gravitational acceleration (9.8 m/s$^2$), *d* is the travel distance (m), *P* is the robot’s power in terms of input voltage (12 V) and input current (2 A), and *v* is the robot’s velocity (between 0.02 and 0.06 m/s). Through trials, the COT for the robot is measured between 95 and 315 for different tail frequencies and robot velocities, with the best COT being at the highest velocity of 6 cm/s and the highest tail beat frequency of 2.3 Hz, indicating more efficient swimming and COT at higher swimming speeds.

## Results

### Reinforcement learning (RL)

**Figure 8:**
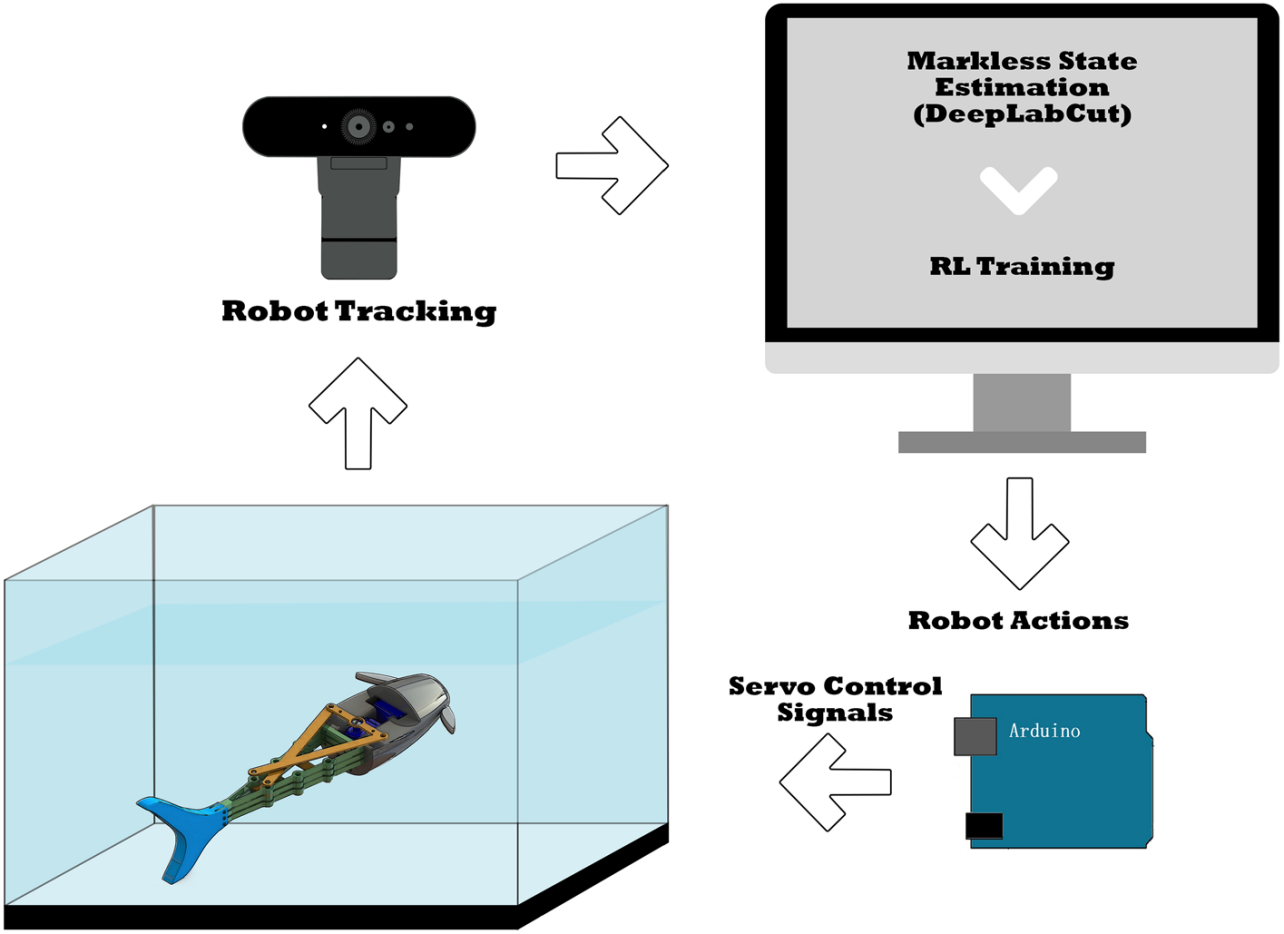
Schematic of the experiment setup.

The main objective of this experiment is to make the robot swim to a certain predetermined goal location in the tank. The setup is shown in Fig. 8. The tank and robot are monitored using a Logitech Brio camera at 60 fps that captures the environment and feeds the frames to DeepLabCut to perform pose estimation. The trained neural network was able to provide accurate pose estimation of the robot with minimal pose loss after the training. In addition, the high capture rate of 60 fps compared to the slow swimming motion of the robot allows the neglect of pose point loss of some frames during the RL training. Moreover, the network outputs a likelihood of the prediction, which gives an indication of occlusion or pose loss of the tracked points. If the likelihood value of a pose point prediction is lower than a certain threshold during the current frame, this pose is discarded and the last known pose with likelihood above the threshold is kept. The likelihood threshold chosen during the RL training is 0.8.

To perform RL training, generally, simulation tools are first used to train the agent, then the learning is transferred to the actual robot. However, due to the complexity of simulating soft materials that exhibit high deformation and the fluid-structure interaction between the robot and environment, the RL training algorithm was implemented directly on the experimental setup. By using stable baselines 3^42^ on top of OpenAI Gym^43^, the RL environment is built by defining the observation and action spaces for the agent.

The actual possible actions for the robot could be described as a continuous space of varying servo speeds and angles, up to the maximum values according to the servo’s specifications. However, having a continuous action space could make the problem more complex to solve for the RL algorithm. Hence, the discretization of the actions would help simplify the task. Thus, the oscillation of the servo is fixed as the maximum travel of the servo, which is $130^\circ$ according to the manufacturer’s specifications. The servo oscillation speed becomes the only variable for the actions. By using the fish swimming analysis as a base, several oscillatory frequencies of the tail were chosen to be applied as the varying speed of the actuation servo motor. These speeds comprise the action space of the robot. A total of 10 actions are defined, ranging between 110 and 200 milliseconds (ms) to perform a tail stroke, with a 10 ms step.

The observation space is comprised of several parameters related to the robot and its environment. First, the x and y positions are obtained from the state estimation performed through DeepLabCut for 3 points on the robot: the head, the servo horn, and the tail. The distances in the x and y directions between the robot’s head and the destined goal point are also added. Finally, a queue of previous actions is appended to the state.

$$\begin{aligned} s_t&= \{p_1(x, \ y)_t, \ p_2(x, \ y)_t, \ p_3(x, \ y)_t, \ \delta x_t, \ \delta y_t, \ a_{t-k}, \ \ldots , \ a_{t-1}\} \text { for last k actions} \\ a_t&= 0, \ \ldots ,\ 9 \end{aligned}$$

where *s* is the observation space. $p_1(x, y), p_2(x, y), p_3(x,y)$ are the x and y coordinates for the head, servo horn, and tail at step *t*, respectively. $\delta x$ and $\delta y$ are the x and y distances between the robot’s head point and the current goal. *a* is the action space consisting of 10 actions from 0 to 9, corresponding to the servo speed ranging from 110 to 200 ms, with an increment of 10 ms. *k* is taken as 100, which is the predefined maximum episode length for this experiment.

Two goals are defined at the two ends of the tank. The robot’s task is to reach the current goal, then the goal changes to the other end once the robot succeeds. To simplify the task, an error tolerance is defined and the robot is considered successful in reaching the target if it swims within a distance of 50 pixels away from the target. The reward function *r* defined to achieve the task is:

2 $$\begin{aligned} r_t = (\alpha \times e^{\frac{-dist_t}{\beta }}) - (\phi \times (dist_t \times i_t)) \end{aligned}$$

where the reward is the exponential of the euclidean distance *dist* between the robot’s head point and the goal, and a penalty term as a factor of the distance and the episode step *i*. $\beta$ is a reward decay factor, $\alpha$ is a reward multiplier, and $\phi$ is a penalty factor. An additional reward is added when the robot reaches the goal point. Since the only terminal state for an episode is reaching the goal with no specific failure state, a maximum limit for steps per episode is defined and the penalty applied to the reward relied on the number of steps elapsed during the episode, increasing as the episode goes longer. The steps limit and the variable factors in the reward function were chosen by trial. Maximum steps per episode, $\alpha$, $\beta$, $\phi$, and goal reward are set as 100, 10, 200, $10^{-5}$, and 200, respectively.

**Figure 9:**
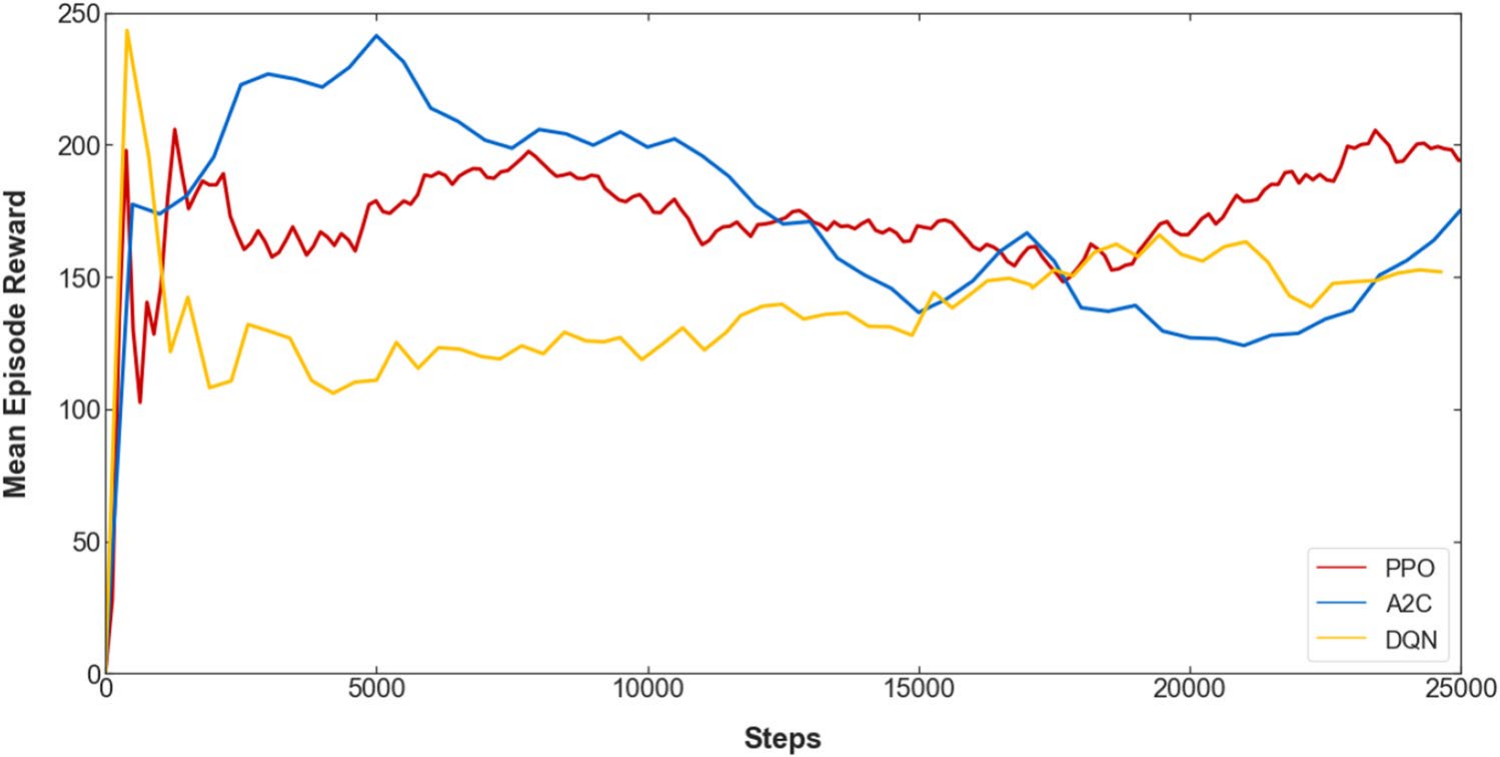
Mean episode reward during the initial training of the three algorithms for 25,000 steps.

**Figure 10:**
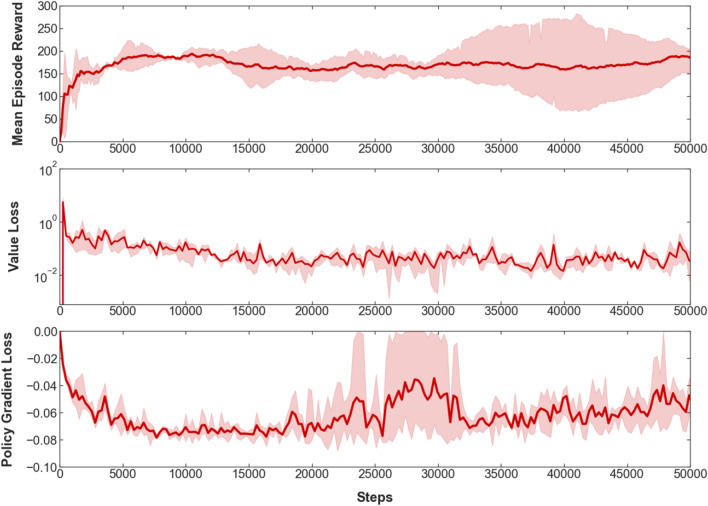
Mean episode reward, and value and policy gradient losses during training of the three PPO agents for 50,000 steps.

To train the robot, three RL algorithms are used to compare their performances. The first two are on-policy algorithms: proximal policy optimization (PPO)^44^ and advantage actor-critic (A2C)^45^, which are policy-gradient methods. The third one is the deep q-network (DQN)^46^, an off-policy value-based method. Performing training on the actual robot is affected by the hardware limitation, such as the durability of the materials used and the inability to operate the servo motor for long periods. Thus, the training was conducted for limited periods, as each algorithm was trained for about 25,000 steps to compare their performance. The mean reward per episode for the three algorithms during the initial training steps is shown in Fig. 9. We can observe that the reward and the robot’s behavior during this initial training are more consistent using PPO, compared to A2C and DQN. Taking into account these results, the PPO algorithm is chosen to be used for further training. Three PPO agents with different random seeds were trained for about 50,000 steps each, with training parameters as shown in Table 1. The agents’ mean episode reward and losses during training are shown in Fig. 10.

**Table 1:** PPO training parameters.

Hyperparameter	Value
Learning rate	3e−4
Discount factor	0.99
GAE	0.95
Clip range	0.2
Value loss coefficient	0.5
Entropy coefficient	0.0
Reward normalization	Yes
Epochs	10
Minibatch size	32
Steps per update	128
Total timesteps per agent	50,000
Agents	3

**Figure 11:**
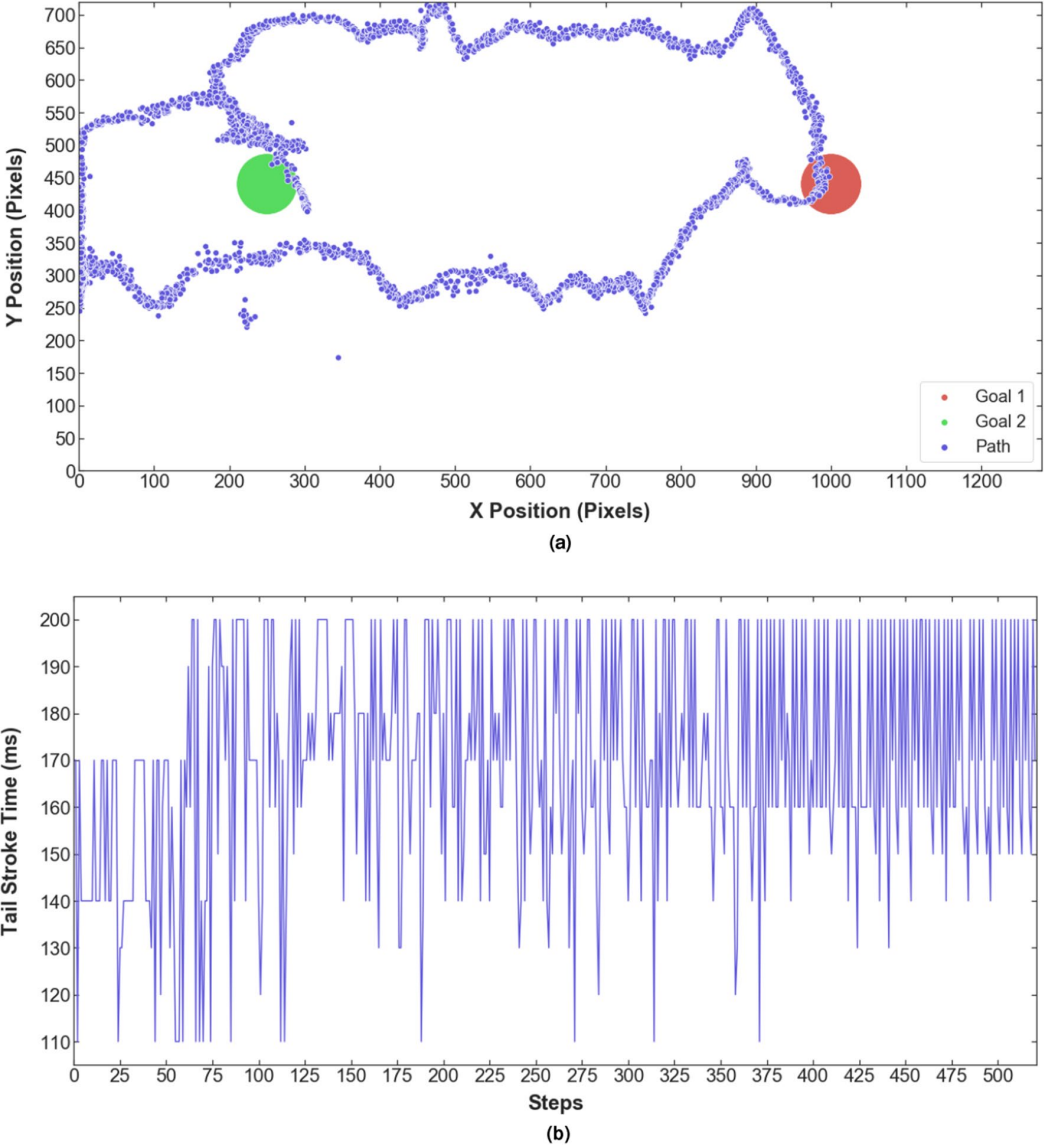
Results of the test run. (**a**) The path taken by the robot to reach the two goals. (**b**) The sequence of actions represented as varying tail stroke time by changing the servo speed.

The best agent is tested on the task to reach the goal within the least amount of steps. Figure 11 shows the path and actuation oscillation frequencies taken by the robot to reach the two defined goals. During the test, the robot starts at a random location in the tank and swims toward the target goal 1. Reaching the first goal rewards the robot and changes the target to goal 2. The robot can change direction and swim toward the second goal, getting another reward and ending the test episode. The robot is considered successful in reaching the target goal if it’s within a 50 pixels distance of it, which is about $95\%$ accuracy.

## Discussion

Developing control algorithms for underwater soft robotics systems is a challenging task due to the non-linear soft body dynamics and the complexity of the fluid-structure interaction in underwater environments. This work explored the use of reinforcement learning as a model-free approach to learning a control policy for a soft-rigid hybrid biomimetic robotic fish. Table 2 shows a comparison between our work and other papers in the literature in terms of modeling and control of soft underwater robots.

**Table 2:** Comparison of modeling and control techniques of underwater soft robots in the literature.

Reference	Type of robot	Modeling techniques	Control/task	Biomimicry
This work	Soft-rigid hybrid robot	Model-free	Reinforcement learning/target goal reaching	Fish
^47^	Rigid robot with compliant part	Model-based: Kirchhoff’s equations and Euler–Bernoulli beam theory	PID heading control	Fish
^48^	Soft-rigid hybrid robot	Model-based: lumped-parameter approach	–	Fish
^49^	Soft-rigid hybrid robot	Model-based: Cosserat rod theory	–	Cephalopod
^50^	Soft-rigid hybrid robot	Model-based: Euler–Bernoulli beam theory	–	Fish
^51^	Soft-rigid hybrid robot	Model-based: pseudorigid-body model (PRBM)	–	Fish
^27^	Rigid robot with compliant part	Model-free	Reinforcement learning/swimming velocity maximization	Cuttlefish
^52^	Soft-rigid hybrid robot	Data-driven lumped-parameter approach	PID amplitude control	Fish
^53^	Soft robot	Model-based: discrete elastic rod method	–	Seastar
^54^	Soft-rigid hybrid robot	Data-driven lumped-parameter approach	–	Octopus
^30^	Soft-rigid hybrid robot	Data-driven model	Reinforcement learning/bipedal underwater walking	Octopus
^28^	Soft-rigid hybrid robot	Model-free	Reinforcement learning/straight line swimming	Eel

In this work, one of the main challenges was running the RL training directly on the robotic hardware. The number of training episodes and the possibility to train multiple agents becomes limited as the training is time-consuming and affects the lifespan, durability, and properties of the soft material, changing its behavior with time. One solution would be the development of an appropriate physics simulator capable of simulating and performing RL on multi-body soft robots in underwater environments, then optimizing the learning through sim2real techniques. Some studies already worked on using accelerated simulators and computational design synthesis to jointly co-optimize the design and control of soft robots^55^, developing data-driven sim2real techniques^56^, or developing spatial and shape grammar in conjunction with using RL and optimization algorithms to co-design morphology and actuation^57,58^. These advances in soft robotics simulation would help advance and improve the training process of soft robots’ RL agents. Another potential solution would be through leveraging the advantages of both model-based and model-free techniques to achieve combined hybrid control techniques with better accuracy and efficiency^59^. Another limitation of this work lies in the actuation method that uses a servo motor to achieve soft body deformation. It introduces a rigid component of significant size in the robot, affecting its softness, and limiting the types of maneuvers it can perform and how it can adapt to its environment. Other types of actuation such as artificial muscles made from dielectric materials or microfluidic actuators could help build more efficient soft biohybrid swimmers^60,61^.

Finally, we believe that solving the control problem in soft robotics comes hand in hand with solving the modeling problem while considering the high dimensionality of these models and their applicability in real-time control. Promising approaches include the exploitation of the robot’s softness through embodied intelligence and morphological computation, the use of reduced-order models alongside robust control, and infinite dimensional control^24^.

## Conclusion

In conclusion, this paper proposed a design for a biomimetic robotic fish with a compliant tail inspired by the Pangasius fish. The robot utilizes a fin ray structure that is made from soft elastic materials and is actuated by a servo motor. The deformation of the soft fin ray tail of the robot fish mimics the undulatory motion of the Pangasius fish during carangiform swimming. The varying undulation frequency of the tail allows the robot to perform underwater locomotion similar to the actual fish. We also investigate the possibility of learning a control policy to teach the robot a certain task, which is reaching a specific target goal in this case. By using reinforcement learning (RL), the robot was able to learn to reach two different goals at opposite locations in the tank. Training the RL algorithm directly on the real prototype eliminates the need to use any sim2real transfer methods. Despite the complexity of the soft robot dynamics, the fluid–structure interaction, and the hydrodynamic forces, the learning process provides good results for the specified task. Training the agent for more steps would possibly allow it to exploit the environment more and learn more complex swimming behavior.

We believe that RL could be similarly implemented on a soft robot with embedded soft actuators, as it has no previous knowledge or model of the system. Changing the actuation method to a soft actuator would change the formulation of the action space. Assuming the consistent performance of the actuator to provide the intended actuation behavior, the RL algorithm should be able to learn a control policy, as has been shown previously in the literature. Further investigation with different actuation and soft robotic systems is intended as the next step.

In future work, we will build on the current advances working to incorporate differentiable simulation and neural network hydrodynamic simulation to allow RL training in simulation and then apply sim2real transfer techniques.

## Methods

No experiments were conducted on the real fish described in the paper, it was only recorded using a Logitech C920 visual monocular camera. It was tracked using a markerless tracking approach based on deep learning computer vision through the animal behavioral analysis library DeepLabCut, as described in the “Pose Estimation” section.

## Supplementary Information

Supplementary Information.

## Data Availability

All data generated or analyzed during this study are included in this published article [and its supplementary information files].
