# Supplementary material for: Design and control of soft biomimetic pangasius fish robot using fin ray effect and reinforcement learning
Source: Sci Rep. 2022 Dec 18;12:21861. doi: 10.1038/s41598-022-26179-x (PMC9760642; doi:10.1038/s41598-022-26179-x)
Supplement: Supplementary file 1 — Supplementary Information. [file 41598_2022_26179_MOESM1_ESM.zip › Supplementary Materials.pdf]

Supplementary Materials:

Design and Control of Soft Biomimetic Pangasius Fish Robot Using Fin Ray Actuator and Reinforcement Learning

Samuel M. Youssef[1,*], MennaAllah Soliman[2], Mahmood A. Saleh[2], Ahmed H. Elsayed[3], and Ahmed G. Radwan[4,5]

[1]Smart Engineering Systems Research Center (SESC), Nile University, Sheikh Zayed City 12588, Egypt.
[2]Bio-Hybrid Soft Robotics Laboratory (BHSRL), Nile University, Sheikh Zayed City 12588, Egypt.
[3]Innovation Hub, Nile University, Sheikh Zayed City 12588, Egypt.
[4]Nanoelectronics Integrated Systems Center (NISC), Nile University, Sheikh Zayed City 12588, Egypt.
[5]Department of Engineering Mathematics and Physics, Cairo University, Giza 12613, Egypt
[*]s.youssef@nu.edu.eg

1. Videos:

Video S1.

Pose estimation of the fish's swimming motion at three different speeds: slow, medium, and fast. The pose estimation was conducted using a webcam and the deep learning markerless pose estimation and behavioral analysis library DeepLabCut. Three points are tracked on the fish: the head, the pectoral fins, and the tail (caudal fin). The plotted swimming trajectories are for points with a prediction likelihood threshold above 80%.

Video S2.

Pose estimation of the untrained robot's swimming motion at ten different speeds before training the reinforcement learning agents. The robot's speed is adjusted by controlling the time the servo takes to perform a single tail stroke. The implemented tail stroke times are in the range of 110 ms to 200 ms, with a 10 ms step interval. The pose estimation was conducted using the same method as mentioned with the fish. The three points that were tracked on the robot are the head, the servo horn, and the tail. The plotted swimming trajectories are for points with a prediction likelihood threshold above 90%.

Video S3.

A successful test run after the RL agent training of the robot. The robot started from an arbitrary position in the tank and succeeded in reaching the first goal, then changed its direction and swam to the second goal. The agent used for this test run is one of the three agents trained using the PPO algorithm, after 50,000 training steps. The plotted trajectory shows the swimming path of the robot during the test run, as captured using the DeepLabCut state estimation with a prediction likelihood threshold above 80%.

2. Data

Data S1.

The results from the pose estimation prediction of the fish's swimming recordings in Video S1.

Data S2.

The results from the pose estimation prediction of the robot's swimming recordings in Video S2.

Data S3.

The mean episode reward for the three RL algorithms (PPO, A2C, and DQN) during the initial training of 25,000 steps, as shown in Figure 8.

Data S4.

The mean episode reward, value loss, and policy gradient loss of the three PPO agents trained for 50,000 steps, as shown in Figure 9.

Data S5.

The state estimation data of the robot's position and its servo actions during the RL trained agent test run in Video S3, describing its swimming path, shown in Figure 10(a), and the servo's sequence of tail stroke times (in ms), shown in Figure 10(b) .
